# Tui Na for painful peripheral neuropathy in people with human immunodeficiency virus: A randomized, double-blind, placebo-controlled trial protocol

**DOI:** 10.3389/fneur.2023.1113834

**Published:** 2023-02-23

**Authors:** Xingmei Zhu, Song Ge, Linda Dune, Chao Yang, Chong Tian, Yong Wang

**Affiliations:** ^1^Yaxin School of Nursing, Wuhan Institute of Design and Science, Wuhan, Hubei, China; ^2^Department of Natural Sciences, University of Houston-Downtown, Houston, TX, United States; ^3^The University of Texas MD Anderson Cancer Center, Houston, TX, United States; ^4^School of Nursing, Huazhong University of Science and Technology, Wuhan, China; ^5^Department of Tuina, Shandong Provincial Hospital Affiliated to Shandong First Medical University, Jinan, Shandong, China

**Keywords:** HIV, neuropathy, massage, pain, randomized controlled trial, traditional Chinese medicine, complementary and alternative medicine

## Abstract

**Background:**

Peripheral neuropathy (PN), including numbness, loss of sensation, paresthesia, a burning sensation, and stabbing pain in extremities, is a common complication in people with human immunodeficiency virus (PHIV). Medications commonly used to treat HIV-related PN are not effective and lead to many side effects. HIV-related PN symptoms may be alleviated or treated with a series of therapeutic Chinese foot massages (TCFM), which are non-invasive and relatively safe. However, relevant studies are lacking.

**Study design:**

This proposed trial is a prospective, two-arm, parallel, double-blinded, randomized controlled trial.

**Aim:**

This proposed trial aims to assess the effectiveness of TCFM on HIV-related PN in people with HIV (PHIV).

**Outcomes:**

The primary outcomes, measured at baseline, end of TCFM/placebo, and twelve weeks after, include (1), lower extremity pain, (2) lower extremity functioning, and (3) health-related quality of life. The secondary outcomes, measured throughout the trial process, include (1) recruitment and completion rate (No. of referred, No. of eligible, No. of enrolled, No. of withdrawals, trial recruitment rate, and trial completion rate), (2) participants' safety (No. and severity of adverse events), (3) treatment adherence (average time of each message session, No. of completed sessions, and No. of missed sessions), and (4) compliance (No. of participants completing the trial following the initial group assignment).

**Sample size:**

An estimated 142 participants in total, or 71 participants in each arm, will be needed for this trial.

**Trial status:**

This trial was registered at ClinicalTrials.gov of the National Institute of Health on Oct 26, 2022 (ClinicalTrials.gov Identifier: NCT05596123). The researchers expect to recruit participants starting in Feb. 2023 and ending in Feb 2025.

## Introduction

More than a million people in the US are infected with the human immunodeficiency virus (HIV). They may suffer from progressive immune system failure and its complications over time due to the retrovirus (1). People with human immunodeficiency virus (PHIV) are nowadays living longer because of the development of antiretroviral therapy (ART) and interventions to increase medication adherence over the years (2, 3). Young PHIV in the US can expect to live into their early 70s with an increased life expectancy of 14.7 years between 2000–2002 and 2006–2007, which is very close to the average lifespan of the general population (4–6). As a result, HIV in the US has gradually evolved from an infectious disease to a chronic condition requiring ongoing self-care and symptom management for a lifetime. PHIV are more likely to experience the neurotoxic side effects of ART due to their potentially longer exposure to ATR with an increased life span (7).

Peripheral neuropathy (PN), the most prevalent neurological symptom in this population, affects about 30–67% of people with PHIV. Although common, PN is often overlooked and misdiagnosed in PHIV (8). HIV-related PN is associated with neurotoxic effects of ART and HIV virus (8, 9). Numbness, loss of sensation, most often in the toes and soles, paresthesia, a burning sensation, and stabbing pain are the major symptoms of HIV-related PN, which most commonly affects feet and less frequently hands (10, 11). There are six and more types of HIV-related PN (8). The type of ART that PHIV use, the stage of their HIV infection, their immune function, and the presence of additional infections affect the type and severity of HIV-related PN symptoms (8, 12). PN compromised PHIV's quality of life (13). However, there is no currently available treatment for HIV-related PN that has been approved by the U.S. Food and Drug Administration. Many PHIV endure PN without using any interventions or medications. For symptom management, some PHIV either no longer take the associated medications or use off-label analgesics, antidepressants, or anti-seizure medications. These medications may cause side effects, including nausea, vomiting, dizziness, and fatigue, which are very common and further disrupt PHIV patients' daily lives. Moreover, these medications only partially alleviate PN symptoms (14). Unfortunately, pharmacological interventions to relieve HIV-related PN are rarely evaluated in research. To our best knowledge, in published studies, only topical capsaicin, smoked medical marijuana, and recombinant human nerve growth factor outperformed placebo in treating HIV-related sensory neuropathy pain (11). To treat PN in PHIV, a combination of pharmacological and nonpharmacological approaches is desired (15). Unfortunately, nonpharmacological interventions on HIV-related PN, however, were the subject of very few existing studies. To our knowledge, only hypnosis (16), spinal cord stimulation (17), lower extremity splinting (18), vibratory stimulus (19), progressive-resisted exercises (20), cognitive behavior therapy (21), and acupuncture/moxibustion (22) have been examined by randomized control trial with the purpose of alleviating HIV-related PN. All the above studies were subject to small sample sizes and methodological limitations. Moreover, only the acupuncture/moxibustion study of the above-mentioned studies demonstrated marginally significant pain reduction compared to the placebos (20). Therefore, non-pharmacological inventions are urgently needed to explore the treatment of HIV-related PN.

Tui Na or therapeutic Chinese massage (TCM) has been used as a therapy in China for more than 5,000 years to 1600–1000 BC (23, 24). Early in TCM history, the technique was described as AnMo (massage) and Dao Yin (physical and breathing exercises), but in current times involves the systematic manual palpation, push-pull, and stroking of acupoints, soft tissues, and muscles by the fingers, hands, elbow, knee, or feet of a therapist (25). Pressure on the foot's and lower leg's muscles and joints will stimulate the somatosensory system by stimulating several receptors and increasing blood flow in the associated areas (26). TCM may assist in removing energy blocks along particular meridians linked to particular diseases or symptoms (24). TCM has been used to alleviate chronic low back pain and neck pain (27, 28), help manage cervical radiculopathy (29), and control hypertension and diabetes (25, 30). A study has found that participants of high-intensity cyclic exercise who also received TCM were significantly more physically fit, better exercise performance, more balanced body composition, and improved physical and mental health (31). Additionally, few adverse events occur when this non-invasive treatment is s if performed by trained professionals (32).

Previous studies with small sample sizes and methodological limitations have provided some evidence that massage could improve PN caused by diabetes (26, 33). To the best of our knowledge, no study has examined the effectiveness of TCM on HIV-related PN. Thus, the aim of this proposed trial to propose a randomized controlled trial that aims to (1) investigate whether a series of 25-min sessions of TCFM (tui na) given six times a week, in comparison to a placebo massage, could help PHIV with lower extremity PN symptoms feel better and function better and (2) evaluate recruitment rate, completion rate, participant safety, treatment adherence, and treatment compliance of such trial.

## Materials and methods

### Ethical approval

The University of Houston-Downtown Committee for the Protection of Human Subjects approved the proposed trial of this protocol after conducting an ethical review. Prior to being enrolled in the trial, participants will be fully informed regarding the possible risks involved, the informed consent process, confidentiality, and secure data storage. Participants will be informed that although TCM is generally safe, mild discomfort, soreness, and slight bruising may appear at or after a session. Severe but very rare adverse events include disc herniation, soft tissue trauma, neurologic compromise, spinal cord injury, vertebral artery dissection, bone fracture, hematoma or hemorrhagic cyst, syncope, cauda equina syndrome, pain, and dislocation (32). Participants will be informed that their decision of whether to participate in this trial will not affect any health or education service they receive at present or in the future. Participants will be guaranteed that they can leave the trial at any time without providing a reason or leading to any consequences. Oral and written informed consent will be obtained from every participant. This trial was registered with ClinicalTrials.gov of the National Institute of Health on Oct 26, 2022 (ClinicalTrials.gov No: NCT05596123).

### Trial design

**Figure 1:**
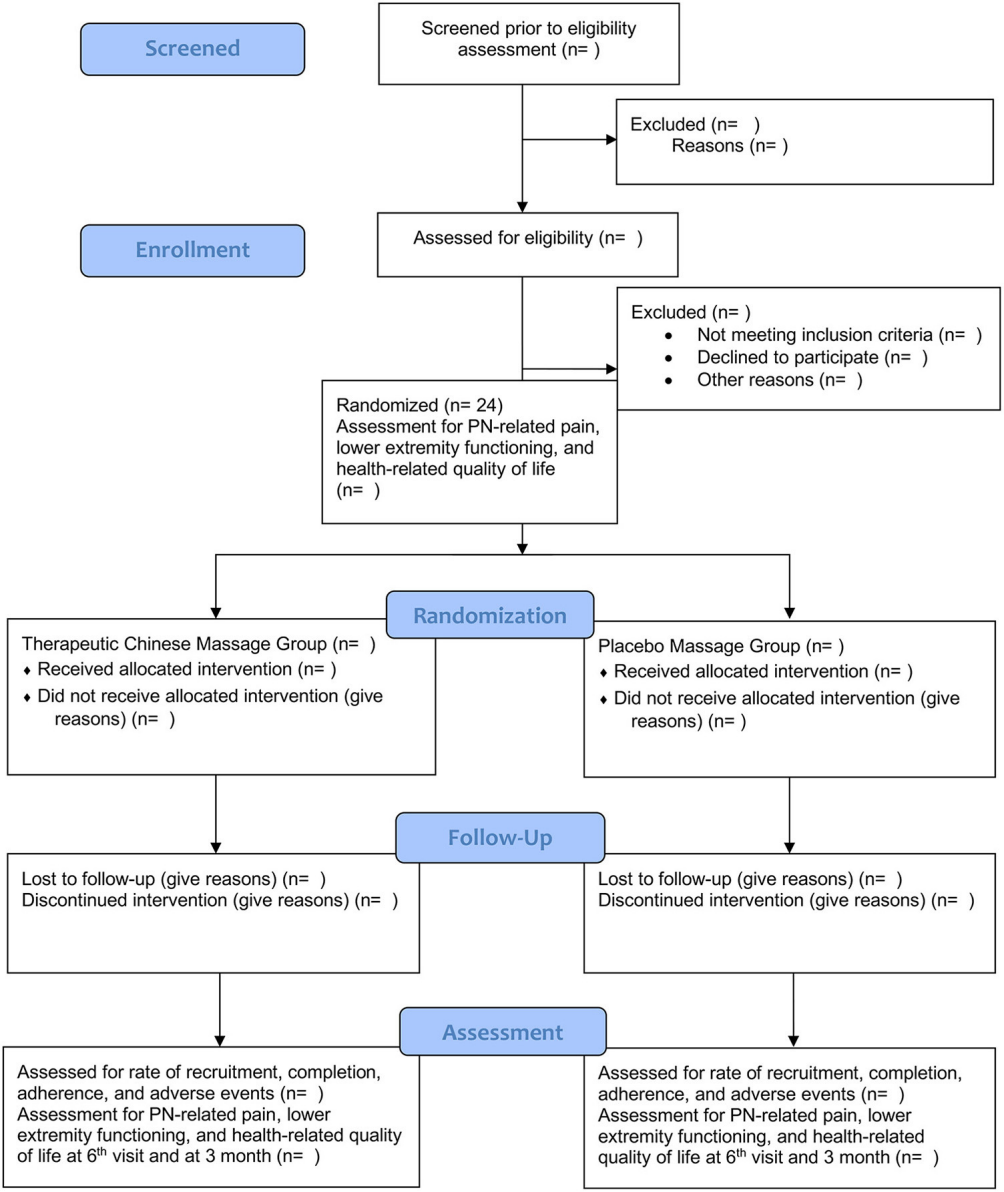
The flow chart of the study.

This proposed trial is a prospective, double-arm, parallel, double-blinded, randomized controlled trial. Participants, the outcome assessors, and the statistician will be unaware of the group assignment. A one-to-one ratio will help randomly assign participants to the TCFM group or the placebo massage group evenly. Six weekly 25-min TCFM sessions will be delivered to participants in the TCFM group. Six weekly 25-min placebo massage sessions will be delivered to participants in the placebo massage group. Depending on the convenience and preference of participants, the TCFM or placebo massage will be conducted either at participants' homes or in a room provided by University of Houston-Downtown. The trial will be reported following the Consolidated Standards of Reporting Trials (CONSORT) Statement (34). Figure 1 shows the enrolment, interventions, and assessment schedule of this proposed trial.

### Study population and eligibility criteria

Participants' inclusion criteria are those who (1) have confirmed HIV diagnosis based on providers' verification with participants' approval; (2) self-report PN-related symptoms in their lower extremity, including sharp, jabbing, throbbing, or burning pain, numbness, decreased sensation to pinprick, prickling or tingling feeling, lack of coordination and falling muscle weakness, and extreme sensitivity to touch (35); (3) are not taking any medications, including pain medicine, to alleviate PN; (4) age 18 years and older; (5) can communicate with researchers in English or Mandarin Chinese; (6) are not pregnant or lactating; (7) are not enrolled in other clinical trials. People who have received non-pharmacological interventions, including any type of massage, to treat their PN symptoms in the past 6 months will be ineligible for participation.

### Recruitment

Two interventionists will be trained by the principal investigator on the recruitment protocol of this trial prior to the beginning of the trial. Flyers, officers' recommendations, sharing posts on Facebook, and the snowball method will be used to recruit PHIV from healthcare facilities at the Texas Medical Center in the US. People interested in the trial are encouraged to get in touch with the PI by phone or email to see if they qualify. Every interested respondent will have a phone or zoom conversation with the PI or the interventionists as an initial screening. The PI or the interventionists will inquire as to whether they experience any of PN symptoms in their lower extremity, such as numbness, decreased sensation to pinprick, prickling or tingling, lack of coordination and falling, muscle weakness, and extreme sensitivity to touch. If they exhibit PN symptoms in their lower extremity, the PI or the interventionists will schedule a time to meet them face-to-face at their home or a room provided by the University of Houston-Downtown, depending on their preferences and convenience. The PI or the interventionists will conduct a complete screening of the person against the inclusion and exclusion criteria when they meet in person, determine whether they are eligible for participation, and obtain informed consent if they are eligible and willing to participate in the trial. The PI, the interventionists, the outcome assessors, and the therapists will all wear facial masks throughout the trial to protect every person they meet.

### Randomization

Using a randomized blocked design function with a block size of four of R statistical software (version 2020) (36), participants will be randomly assigned to the TCFM or the placebo massage group in a one-to-one ratio. This will guarantee that up until the desired sample size is reached, each group will have roughly equal size of participants. The therapist will be informed of the group assignment after a participant gives consent to participate in the trial and the baseline data are collected. Since each participant's information will be de-identified, a unique ID number will be generated to represent the identity of that participant. The confidentiality of all participants' information will be maintained throughout and after the trial. Access will only be permitted to those who are authorized.

### Sample size

The sample size per arm is calculated using longpower R package (a package for longitudinal data sample size calculation) (37, 38). For a longitudinal design with two arms (with allocation ratio = 1:1) focusing on the difference in change from baseline at the last visit in SF-MPQ-2 total score, with a power of 0.8 and significance level of 0.05 (two-sided), a dropout rate of 25% at post-baseline visits in both arms, a compound symmetric within-individual correlation matrix with an intraclass correlation coefficient of 0.8 in both arms, a standard deviation of 2 for change in score from baseline at last visit (assuming the same in both arms), and effect size of 1, the sample size per arm is 71 (142 in total).

### Blinding

While participants, the statistician, and the outcome assessors will be blinded, the PI and the therapists will be aware of participants' group assignment. This trial is, therefore, double blinded. However, it is likely that participants will have the right guess about the type of massage they have received.

### The TCFM (Tui Na)

Participants in the TCFM group will receive 6 weekly 25-min TCFM sessions provided by a therapist. The therapist will begin each session with an assessment of the legs and toes of the affected extremity for broken skin and lesions that would identify areas at risk for irritation during the massage and need to be avoided. The participant will be positioned with support to their foot and legs, with the sole of the foot plantar flexed and the therapist directly in alignment with the foot. The therapist will serially perform the following four steps for each TCFM session.

#### Step one: Releasing the channel sinews

One finger-pressing massage and vibration will be applied along the lower extremity points Gall Bladder Channel (39) points Qiuxu (GB40), Juegu (GB39), Yangfu (GB38), Guangming (GB37), Waigu (GB36), Yangjiao (GB 35), and Yanglingquan (GB34) using the thumb tip of the dominant hand of the therapist. All pressing and vibration maneuvers will be repeated for three rotations along the above identified GB channel of 60 s per point with the same repeated sequence along the channel. The vibrating massage will deliver 50 strokes for 30 s per point to release the sinews.

#### Step two: Stimulating the AhShi, along with breaking up and dispersion of stagnations

During the finger-pressing massage, the therapist will identify any sensitive areas, trigger points, contracted muscle areas, and nodules. As those areas are identified during one finger-pressing procedure, additional time will be spent to elicit a therapeutic response of change in sensation. The AhShi points will be treated with deeper penetrating pressure to surrounding the dragon (a local treatment using intense energy around the affected area) using vibration. The stagnation will be further dispersed with consistent pressure to the center of AhShi with the whorled surface of the thumb.

#### Step three: Moving and invigorating the flow of Qi and blood

A rolling technique will be applied for three passes from the heel to the toes to invigorate Qi flow. The rolling will be concluded with pressing and vibrating manipulation with the interphalangeal joint of the middle finger of the therapist to the participant's Yongquan (KI 1) acupoint. Step 3 will be repeated three times on the plantar surface of the foot.

#### Step four: Expelling and clearing pathogenic factors

GB 40, Zulinqi (GB 41), and the sole of the foot will be massaged with major thenar kneading manipulations for three passes beginning with soft then more penetrating kneading movements from ankle GB 40, 41 to the plantar surface of the foot. Further treatment of the foot will include shaking manipulation and rotation to the ankle. To further generate Qi flow and clear pathogenic factors, toe rotation and pulling will be followed by rubbing of Shangqiu (SP 5) until the participant reports a warm sensation over the medial malleolus of the ankle.

### The placebo massages

The same therapist will provide 6 weekly 25-min placebo massage sessions to participants in the placebo group. These sessions will include an assessment of the affected extremity's legs and toes for lesions and broken skin to avoid them during the massage. The provider will perform an open hand technique of gentle foot and toe rubbing without any point stimulation or other TCFM techniques.

### The TCFM/placebo massage therapist

Two massage therapists in this trial will have Diplomate in Asian Body Works and a certified Chinese massage therapist. The two must have more than 2 years of clinical experience in this field. The two therapists will deliver both TCFM and placebo massage to all participants in both groups. The two therapists will go through a 4-h training with the PI before the commencement of the trial to familiarize the therapists with the trial protocol. After the training, the two therapists must pass an exam in which they must verbally recite the protocol and physically demonstrate each technique to the PI.

### Safety

TCFM is generally safe with the possibility of rare adverse events that might include mild discomfort, soreness, and slight bruising during or after a session (32). The safety measures will include assessment and treatment with monitoring of responses that enable the therapist to adapt the treatment to patient needs. Assessment will include skin inspection of the legs, feet, and toes for lesions, indurations, or damage. The therapist will avoid massaging the areas of skin breakage. Participants will be encouraged to verbalize experiences of increased pain, increased sensitivity in their feet, and additional discomforts of tenderness, nausea, sweating, skin rashes, or extreme fatigue. If the participants complain of increasing pain the therapist will provide more gentle therapy. The therapist will assess any unfavorable participant reactions. The therapist will carefully document these events in the case report form. To avoid the spread of infectious agents, The massage therapist will don latex or nonlatex (for those who are allergic to latex) gloves while performing the massage and perform hand hygiene after each session.

### Adherence

The participant will be reminded about their appointment by text message 1 day before their scheduled session to improve adherence to the trial. Participants' parking will be reimbursed. Subway tokens will be provided. If a participant has difficulty coming to the site, the researcher and the therapist will go to his/her home.

### Data collection

Two outcome assessors will be trained with the trial data collection protocol by the PI prior to any data collection.

#### Sociodemographic, health, and lifestyle information

Participants' sociodemographic, health, and lifestyle information will be collected using a standardized self-report questionnaire designed for this trial. Information will be collected at the first visit *via* an iPad or paper format before randomization occurs. The questions include age (years), gender (male or female), ethnicity (Mexican Americans, other Hispanics, non-Hispanic White, or non-Hispanic Black), education (below high school, high school graduate, or some college or above), domestic partner status (living with a partner or not), employment status (employed or not), alcohol use (number of standard drinks per day), smoking status (never smokers, former smokers, and current smokers), body mass index, and depressive symptoms. One standard drink contains 14 grams of pure alcohol (40). The Patient Health Questionnaire (PHQ-9) score will be used to indicate depressive symptoms (41). If the participants cannot read the questions by themselves, the interventionist will read the questions to them. Participants' height and weight will be measured for the calculation of body mass index.

#### Primary outcome

PN-related pain, lower extremity functioning, and health-related quality of life will be collected at the first visit after consenting before randomization (baseline). This information will be collected again at their sixth visit after the TCFM/placebo massage (immediate impact) and 12 weeks after the competition of the trial (long-term effect).

1) The Short Form-McGill Pain Questionnaire-2 (SF-MPQ-2) will be used to measure PN-related pain (42). SFMPQ-2, consisting of 22 items from the McGill Pain Questionnaire (MPQ), can reflect both neuropathic and non-neuropathic pain. Each descriptor is ranked from 0 to 10 (0 indicates none; one indicates mild; ten indicates the worst pain possible). Total pain is the average of all 22 items, ranging from 0 to 10. The correlation of SFMPQ-2 with MPQ has been demonstrated.
2) Lower extremity functioning will be measured by the Lower Extremity Functional Scale (LEFS) (score range 0–100) (43). A higher score indicates better lower extremity functioning. The LEFS has strong tes*t-*retest reliability, cross-sectional construct validity, and internal reliability (44). It was demonstrated that it could accurately identify changes in lower extremity musculoskeletal conditions in patients that are clinically significant (45).
3) Health-related quality of life will be measured by the Medical Outcomes Study Questionnaire Short Form 36 Health Survey (SF-36) (score range 0–100) (46). A lower score indicates more disability, and a higher score indicates less disability. According to prior studies, the SF-36's reliability score was higher than 0.80 (46, 47).

The secondary outcomes include (1) recruitment and completion rate (No. of referred, No. of eligible, No. of enrolled, No. of withdrawals, trial recruitment rate, and trial completion rate), (2) participant safety (No. and severity of adverse events), (3) treatment adherence (average time of each message session, No. of completed sessions, and No. of missed sessions) and compliance (No. of participants completing the trial following the initial group assignment). Adverse events will be recorded by the therapists in the notes. A published systematic review of the negative effects of massage therapy in the context of pain found the following adverse but rare events which the therapists will be instructed to watch out for, including disc herniation, soft tissue trauma, neurologic compromise, spinal cord injury, vertebral artery dissection, bone fracture, hematoma or hemorrhagic cyst, syncope, cauda equina syndrome, pain, and dislocation (32).

### Data management

The collected data will be password-protected in a cabinet. The cabinet's passcode will only be known by the PI. The research team will take precautions to keep the data private before, during, and after the trial. No individual's details will be revealed.

### Protocol amendments

To determine whether the protocol needs to be revised, the research team will conduct a review. University of Houston-Downtown Committee for the Protection of Human Subjects will receive the modified protocol, and the participants will be notified as well.

### Statistical analysis

We will summarize the characteristics of the participants at baseline by treatment arm using descriptive statistics. Specifically, we will use mean (standard deviation) for normal (or roughly normal) continuous data, and medians (25th percentile, 75th percentile) for non-normal continuous data, and frequency (percentages) for categorical data. The two-sample *t-*test and chi-square test (or Fisher's exact test, as appropriate) will be used to examine differences by the two arms at baseline.

The primary outcome will be assessed according to intention-to-treat (48) principles. We will use a mixed model for repeated measures (MMRM) (49) to model the change from baseline at each post*-*baseline visit in two arms, with each of the primary outcomes as dependent variable. For each of the outcome, using MMRM, we will compare the difference between the treatment vs. placebo arm in change in mean response from baseline at last visit. The likelihood-based MMRM can handle missingness in the responses when the responses are missing at random (50).

For the secondary outcome, we will calculate rates of recruitment (defined as the number of consented individuals divided by the number of eligible individuals), completion (defined as the percentage of completion at baseline and follow-up visits), adherence (defined as participants' completed sessions divided by the number of offered sessions), and adverse events (in two forms, total number and number per participant).

The statistician is not part of the research team and is not aware of the group assignment. All analyses will be performed using R statistical software (version 2020) (36). A two-sided *P*-value of 0.05 or less will be considered statistical significance.

## Discussion

One of the most prevalent neurological complications associated with PHIV is PN. It has a negative impact on the disturbed lives of PHIV, a vulnerable and marginalized population. Safe, effective, and non-harmful non-pharmaceutical interventions are highly desirable to alleviate and treat PN symptoms in PHIV because there is no FDA-approved treatment for this condition. Surprisingly, research studies on non-pharmacological treatments for HIV-related PN are scarce. To the best of our knowledge, there are only seven non-pharmacological intervention studies on pharmacologic and non-pharmacologic treatments for HIV-related neuropathy pain, and the total number of participants in these seven studies is 742 (51). Our proposed trial will be, to the best of our knowledge, the first one to investigate the efficacy of TCM on HIV-related PN. Thus, it will make an important contribution to research and offer evidence on a possible, low-risk, noninvasive, non-pharmacological intervention to address HIV-related PN, an overlooked issue in PHIV. Researchers in a systematic review have found that there are very few serious adverse events related to massage therapies in general and massage related to pain (32). Thus, TCFM is a promisingly effective and safe intervention to treat HIV-related PN.

Based on traditional Chinese medicine, a person's health is achieved when his or her body's Qi (life force or vital energy) is balanced and unhindered. Blood flow is impeded when Qi is blocked, and pain may develop in the nearby area. The purpose of TCFM is to promote blood circulation and remove energetic blocks. As a result, Qi stagnation and related pain can be removed (25). Moreover, studies revealed that massage therapy could reduce inflammation and promote mitochondrial biogenesis that repairs damaged muscles (52).

This proposed trial has several major advantages. This trial offers a thorough, written research protocol that is transparent and reproducible. The methodology is written in detail and allows reproducibility. Moreover, several crucial HIV-related health outcomes are evaluated. Furthermore, the placebo massage is carefully designed with the intention of not activating acupuncture points or involving techniques of traditional Chinese massage. Thus, the results of this trial will add valuable evidence and implications to incorporating complementary medicine for HIV-related PN, a prevalent but overlooked issue in PHIV. To ensure consistency in the delivery of the intervention and placebo, the same two therapists will administer the TCFM and the placebo massage.

The main limitation of this proposed trial is that although blinded, participants will possibly realize the type of massage they have received. However, all massage-related studies are subject to this methodological flaw. A control group is still important to account for placebo effects (53). After the trial is completed, the therapists will instruct the participants on how to support their health and adopt lifestyle changes that will reenergize and clear pathogenic factors from the channel for long-term health benefits. Participants will be given instructions by the therapists on how to perform at home, convenient toe exercises, including kneading massage to the foot, specifically to the locations of GB 40 and 41, and rolling manipulation to the sole of the foot with a provided racquetball for pressure to KI 1.

In short, this proposed trial will provide important research evidence and clinical implications for the effectiveness of TCM in reducing HIV-related PN. We intend to share the research results at national and international academic conferences as well as regional HIV community events. We will also disseminate the results in peer-reviewed journals as open-access publications.

## Trial status

This trial was registered with ClinicalTrials.gov of the National Institute of Health on Oct 26, 2022 (ClinicalTrials.gov No: NCT05596123). The researchers expect to recruit participants starting in Feb. 2023 and ending in Feb 2025.

## Ethics statement

The University of Houston-Downtown Committee for the Protection of Human Subjects approved the proposed trial of this protocol after conducting an ethical review. Oral and written informed consent will be obtained from every participant.

## Author contributions

XZ, SG, YW, and CT drafted the manuscript. LD wrote the section about the intervention. CY wrote the section about statistical analysis. All authors revised the manuscript, provided their expertise, read, and approved the final manuscript.
